# Prognostic and diagnostic value of eosinopenia, C-reactive protein, procalcitonin, and circulating cell-free DNA in critically ill patients admitted with suspicion of sepsis

**DOI:** 10.1186/cc13908

**Published:** 2014-06-05

**Authors:** Jose Garnacho-Montero, María J Huici-Moreno, Antonio Gutiérrez-Pizarraya, Isabel López, Juan Antonio Márquez-Vácaro, Hada Macher, Juan Manuel Guerrero, Antonio Puppo-Moreno

**Affiliations:** 1 Unidad Clínica de Cuidados Críticos y Urgencias, Hospital Universitario Virgen del Rocío, Avd Manuel Siurot s/n., 41013 Sevilla, Spain; 2 Instituto de Biomedicina de Sevilla (IBiS), Hospital Universitario Virgen del Rocío / CSIC / Universidad de Sevilla, Avd Manuel Siurot s/n., 41013 Sevilla, Spain; 3 Red Española de Investigación en Patología Infecciosa (REIPI), Hospital Universitario Virgen del Rocío, Avd Manuel Siurot s/n., 41013 Sevilla, Spain; 4 Unidad de Gestión Clínica de Bioquímica Clínica, Hospital Universitario Virgen del Rocío, Avd Manuel Siurot s/n., 41013 Sevilla, Spain; 5 Unidad de Gestión Clínica de Enfermedades Infecciosas, Microbiología y Medicina Preventiva, Hospital Universitario Virgen del Rocío, Avd Manuel Siurot s/n., 41013 Sevilla, Spain

## Abstract

**Introduction:**

The aims of this study were to assess the reliability of circulating cell-free DNA (cf-DNA) concentrations, compared with C-reactive protein (CRP), procalcitonin (PCT) and eosinophil count, in the diagnosis of infections in patients with systemic inflammatory response syndrome (SIRS) and their prognostic values in a cohort of critically ill patients.

**Methods:**

We conducted a prospective cohort study in a medical-surgical intensive care unit of a university hospital. Eosinophil count and concentrations of cf-DNA, CRP, and PCT were measured in patients who fulfilled SIRS criteria at admission to the intensive care unit (ICU) and a second determination 24 hours later. DNA levels were determined by a PCR method using primers for the human beta-haemoglobin gene.

**Results:**

One hundred and sixty consecutive patients were included: 43 SIRS without sepsis and 117 with sepsis. Levels of CRP and PCT, but not cf-DNA or eosinophil count, were significantly higher in patients with sepsis than in SIRS-no sepsis group on days 1 and 2. PCT on day 1 achieves the best area under the curve (AUC) for sepsis diagnosis (0.87; 95% confidence interval = 0.81-0.94). Levels of cf-DNA do not predict outcome and the accuracy of these biomarkers for mortality prediction was lower than that shown by APACHE II score. PCT decreases significantly from day 1 to day 2 in survivors in the entire cohort and in patients with sepsis without significant changes in the other biomarkers.

**Conclusions:**

Our data do not support the clinical utility of cf-DNA measurement in critical care patients with SIRS. PCT is of value especially for infection identification in patients with SIRS at admission to the ICU.

## Introduction

One of the most frequent problems in the ICU is actually differentiating the inflammatory response from an infective process. Clinical and standard laboratory tests are not very helpful because most critically ill patients develop some degree of inflammatory response, whether or not they have sepsis. Predicting the outcome of intensive care patients is also of particular transcendence to ensure efficient use of hospital resources. Numerous biomarkers have been evaluated to predict mortality in critically ill patients, although none have proved entirely useful. The majority of these biomarkers have also been assessed as a marker of underlying infection in systemic inflammatory response syndrome (SIRS).

C-reactive protein (CRP) and procalcitonin (PCT) are currently the most frequently used biomarkers in clinical practice [1]. PCT is considered to have a higher capacity to diagnose sepsis than CRP [2-4]. Eosinopenia has also been proposed as a marker that may help to differentiate sepsis-related conditions from other causes of SIRS [5]. The usefulness of eosinopenia as predictor of outcome in critically ill patients has also been reported [6]. Eosinopenia is an interesting biomarker because the eosinophil count is always measured in clinical practice and the additional costs would therefore be negligible.

Circulating cell-free DNA (cf-DNA) has recently received growing attention and has been studied in various chronic and acute disorders [7-9]. cf-DNA fragments are small acellular double-stranded molecules with a lower molecular weight than genomic DNA circulating in peripheral blood. Although the origin of cf-DNA has not been completely elucidated, DNA fragments released by apoptotic cells are considered the potential source of this type of DNA [10]. High cf-DNA levels have been reported in severe sepsis and cf-DNA has been proposed as a prognostic marker in patients suffering from sepsis [11]. Moreover, in critically ill patients, infection is associated with higher cf-DNA concentrations [12].

We set out to assess the performance of cf-DNA as a potential biomarker to differentiate SIRS without infection from sepsis. We compared cf-DNA with the two more frequently used biomarkers (CRP and PCT) as well as with the presence of eosinopenia. In addition, we analyzed the reliability of these biomarkers to predict mortality in the entire cohort and in the subgroup of patients with sepsis compared with severity scales at admission.

## Methods

### Setting

This prospective study was carried out in the ICU of the Hospital Virgen del Rocío from July 2010 to June 2011. This ICU is a 40-bed medico-surgical unit in a large university hospital. The Institutional Review Board of the Hospital Virgen del Rocio approved this protocol. Written informed consent was obtained from the patient or the next of kin before inclusion in this study.

### Study design

All adult patients meeting criteria for SIRS on admission to the ICU were enrolled. The diagnosis of SIRS, severe sepsis, and septic shock was established according to the definitions of the American College of Chest Physicians consensus conference [13]. All patients received standard supportive treatment following recommendations of the Surviving Sepsis Campaign released in 2008 [14]. Patients with noncure malignancies [8] and acute myocardial infarction [7] in the last month were excluded from this study. The patients were classified as SIRS or sepsis by two researchers unaware of the biomarker levels.

At ICU admission, severity of the illness was evaluated by the Acute Physiology and Chronic Health Evaluation (APACHE) II score, considering the worst data point for the first 24 hours in the ICU [15]. Failure of organs and severity of multiple organ dysfunction syndrome was assessed by the Sequential Organ Failure Assessment (SOFA) scale [16].

The first blood sample was drawn into a lithium heparin and ethylenediamine tetraacetic acid tube as soon as possible after the patient was admitted to the ICU and written informed consent had been obtained. The second blood sample was collected the following day, 24 hours after the first sample. The serum fraction was separated by centrifugation at 1,000 × *g* for 5 minutes and these samples were stored at -20°C. cf-DNA and PCT were measured in this frozen fraction. In contrast, the eosinophil count and CRP were determined at the moment of separation.

The white blood cell and eosinophil counts were measured by the Coulter hematology analyzer (Beckman Coulter, Brea, CA, USA). The detection limit of the eosinophil count was 10 cells/mm^3^. CRP was measured by an immunoturbidimetric assay on a Modularw P (Roche Diagnostics, GmbH, Mannheim, Germany) chemistry analyzer. The reference limit for this method is less than 0.5 mg/dl. PCT was measured by an immunofluorescent assay using the BRAHMS PCT kit (Roche, Zurich, Switzerland) following the manufacturer’s protocols. The lower detection limit for this method is 0.05 ng/ml.

DNA from 400 μl serum samples was extracted with the automatized ManNa Pure Compact Instrument (Roche Diagnostics, Basel, Switzerland) using the Magna Pure Compact Nucleic Acid Isolation Kit I, according to the Total NA Plasma 100 400 V3 1 protocol. DNA is eluted in a final volume of 50 μl and frozen at -20°C until polymerase chain reaction (PCR) quantification.

Serum DNA was measured using a real-time quantitative PCR assay for the β-globin gene. The quantitative PCR analysis was performed using a Light-Cycler 480 Real-Time PCR instrument (Roche Diagnostics, Basel) by the 5′ nuclease assay (Taqman assay). Two microliters of DNA were amplified in a final volume of 20 μl using the LC480 ProbesMaster Kit (Roche Diagnostics, Basel) according to the manufacturer’s instructions. The β-globin Taqman system consisted of the amplification primers β-globin-354 F (5′-GTG CAC CTG ACT CCT GAG GAG A-3′) and β-globin-455R (5′-CCT TGA TAC CAA CCT GCC CAG-3′) and a dual-labeled fluorescent probe β-globin-402 T (5′-(FAM) TCT GGC CAA GTT TCA ACT CTG CTC GCT (TAMRA)-3′) at 95°C for 5 minutes and at 62°C for 20 minutes for 48 cycles. The final size of the amplicon was 102 base pairs. Results are expressed as the genome equivalent (GE) per milliliter (1 GE = 6.6 pg DNA). cf-DNA was also measured in 10 healthy adults (control group).

### Statistical analysis

Descriptive results of continuous variables were expressed as the mean (standard error) or median (interquartile range) depending of the normality of their distribution. Comparisons between two independent continuous variables were analyzed by *t* test or Mann–Whitney *U* test. For comparisons among more than two continuous variables, an analysis of variance test was performed, followed by the least significant difference test for post-hoc intragroup analysis. A chi-square test was carried out to compare proportions.

Receiver operating characteristic curves and the respective areas under the curves (AUCs) were calculated for APACHE II and SOFA scores and leukocyte, eosinophil, CRP, cf-DNA and procalcitonin levels. Statistical comparisons of the AUC for each parameter were performed with the DeLong test [17] by the MedCalc software 12.7.0 trial version (MedCalc Software bvba, Belgium). The sensitivity, specificity, negative predicted value and positive predicted value were calculated based on the respective cutoff values, which were determined using the Youden index:

$$J=\max\left(\mathrm{sensitivity}+\mathrm{specificity}-1\right)$$

All statistical procedures were performed using SPSS 19.0 statistical software (SPSS, Chicago, IL, USA).

## Results

A total of 163 patients fulfilling the inclusion criteria were entered in this study. In all of these patients, a minimum of two SIRS criteria was present at admission to the ICU. Both researchers did not achieve agreement in three cases, which were excluded. Sepsis was diagnosed in 117 patients (44 with severe sepsis and 73 with septic shock) and 43 patients presented SIRS without infection (no-sepsis-SIRS). In patients with no-sepsis-SIRS, all cultures were negative.

The admission diagnoses of the 43 patients with SIRS but without infection were acute respiratory failure (*n* = 13), early postoperative course of abdominal surgery (*n* = 10), congestive heart failure (*n* = 8), acute pancreatitis (*n* = 8), multiple trauma (*n* = 2) and febrile syndrome in connective tissue disease (*n* = 2).

In patients with sepsis, the most frequent sources of infection were abdominal (*n* = 53) followed by the lung (*n* = 25) and urologic (*n* = 14). Bacteremia was detected in 36/117 (30.8%) patients. Microbiological documentation was achieved in 98/117 patients: Gram-negative infection (*n* = 49), Gram-positive infection (*n* = 34), and polymicrobial infection (*n* = 15).

The mean time elapsed from ICU admission to blood collection for the first determination of biomarkers was 6 hours without differences between patients with sepsis and with no-sepsis-SIRS. The median cf-DNA concentration was 5,265 GE/ml (interquartile range 5,377 GE/ml) on day 1 and 4,895 GE/ml (interquartile range 5,612.5 GE/ml) 24 hours later. The median cf-DNA level in the control group was 283.5 GE/ml (interquartile range 208 GE/ml).

### Entire cohort

#### *Diagnostic value*

Levels of CRP and PCT on day 1 were significantly higher in patients with sepsis than in the no-sepsis-SIRS group (Table 1). This difference persisted on day 2. Conversely, the eosinophil count and cf-DNA on days 1 and 2 did not significantly differ in these two groups. The rate of eosinophil count below the detection breakpoint was higher in patients without infection than in sepsis patients, although this difference was not statistically significant (18/43 (42.9%) vs*.* 40/117 (34.2%); *P* = 0.317). Regarding severity scales, only the SOFA score was significantly higher in sepsis patients than in patients with SIRS without infection.

**Table 1:** Baseline characteristics of the inception cohort

	**Total cohort (*n*** **= 160)**	**SIRS patients (*n*** **= 43)**	**Sepsis patients (*n*** **= 117)**	***P *** **value**^**a**^
**Demographic variables**				
Gender (female)	81 (50.6)	29 (67.4)	52 (44.4)	0.010
Age	63 (51 to 74)	63 (48 to 75)	63 (52 to 73)	0.846
**Underlying disease**				
Diabetes	35 (21.9)	12 (27.9)	23 (19.7)	0.263
Cirrhosis	6 (3.8)	1 (2.3)	5 (4.3)	0.565
ESRD	12 (7.5)	2 (4.7)	10 (8.5)	0.407
Immunosuppression	20 (12.5)	16 (13.7)	4 (9.3)	0.458
Cancer	20 (12.5)	5 (11.6)	15 (12.8)	0.840
Chronic cardiac insufficiency	11 (6.9)	4 (9.3)	7 (6)	0.462
COPD	22 (13.8)	6 (14)	16 (13.7)	0.964
**SIRS**				
Two SIRS criteria	41 (25.6)	12 (27.9)	29 (24.8)	0.036
Three SIRS criteria	57 (35.6)	21 (48.8)	36 (30.8)	
Four SIRS criteria	62 (38.8)	10 (23.3)	52 (44.4)	
**Severity scores**				
APACHE II	17 (12 to 24)	16 (11 to 22)	17 (13 to 24)	0.057
SOFA	7 (4 to 11)	4 (1 to 9)	8 (5 to 11)	<0.001
Worst SOFA	8 (5 to 13)	5 (2 to 9)	9 (6 to 13)	<0.001
**Laboratory parameters**^**b**^				
Leukocytes day 1	13,130 (10,185)	12,780 (8,690)	13,160 (11,280)	0.079
Leukocytes day 2	14,700 (8,650)	12,910 (8,260)	15,395 (7,903)	0.056
Eosinophils day 1	55.6 (11.2)	38.3 (10.6)	61.8 (14.7)	0.434
Eosinophils day 2	91.1 (15.6)	95.6 (19.8)	78 (21.1)	0.533
CRP day 1	26.56 (21.91)	19.5 (25.52)	28.39 (20.98)	0.002
CRP day 2	28.85 (19.66)	23.39 (27.21)	30.2 (19.17)	0.003
PCT day 1	7.81 (34.91)	0.73 (1.49)	13.57 (45.87)	<0.001
PCT day 2	4.82 (20.56)	0.97 (2.58)	10.35 (25.62)	<0.001
cf-DNA day 1	5,265 (5,377)	4,170 (4,590)	5,770 (5,420)	0.081
cf-DNA day 2	4,895 (5,612.5)	4,490 (5,390)	5,090 (5,785)	0.417
cf-DNA day 1, without ARF^c^	5,050 (5,130)	4,105 (4,502)	5,760 (5,240)	0.106
cf-DNA day 2, without ARF^c^	4,960 (5,410)	3,700 (5,210)	5,410 (5,482)	0.077
**Outcomes**				
Length of stay on ICU	7.5 (4 to 14)	6 (3 to 14)	8 (4 to 15)	0.420
ICU mortality	45 (28.1)	13 (30.2)	32 (27.4)	0.719
Hospital mortality	57 (35.6)	16 (37.2)	41 (35)	0.800

Data presented as *n* (%) or median (interquartile range). APACHE, Acute Physiology and Chronic Health Evaluation; ARF, acute renal failure; cf-DNA, cell-free DNA; COPD, chronic obstructive pulmonary disease; CRP, C-reactive protein; ESRD, end-stage renal disease; PCT, procalcitonin; SIRS, systemic inflammatory response syndrome; SOFA, Sequential Organ Failure Assessment. ^a^*P* values for comparisons between SIRS and septic groups. ^b^For comparison, nonparametric tests were used. ^c^ARF defined as renal SOFA ≥3 (*n* = 34).

**Figure 1:**
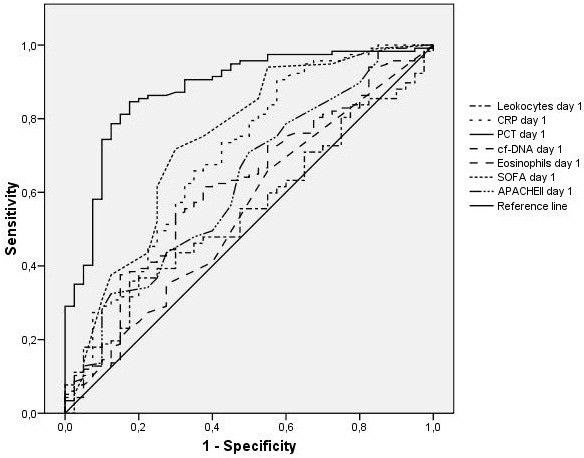
**Receiver-operating characteristic curves in the total cohort for various biomarker cutoff levels.** Receiver-operating characteristic curves in the total cohort for cutoff levels of leukocytes, C-reactive protein (CRP), procalcitonin (PCT), cell-free DNA (cf-DNA) and eosinophils and Sequential Organ Failure Assessment (SOFA) and Acute Physiology and Chronic Health Evaluation (APACHE) II scores in differentiating between the presence and absence of sepsis at admission. Areas under the receiver-operating characteristic curves: leukocytes, 0.55 (95% confidence interval (CI), 0.45 to 0.64); CRP, 0.69 (95% CI, 0.59 to 0.79); PCT, 0.87 (95% CI, 0.81 to 0.94); cf-DNA, 0.51 (95% CI, 0.61 to 0.71); eosinophils, 0.54 (95% CI, 0.44 to 0.65); SOFA score, 0.74 (95% CI, 0.64 to 0.83); and APACHE II score, 0.62 (95% CI, 0.51 to 0.73).

Table 2 summarizes the best cutoff values for the biomarkers calculated using the Youden index and their predictive accuracy for the specific diagnoses of SIRS without infection and sepsis. The AUCs for all biomarkers as predictors of sepsis are shown in Figure 1. On day 1, PCT presents the best AUC (0.87 (0.81 to 0.94)), which is significantly higher than the AUC for the rest of variables (*P* < 0.01). The AUC of the receiver operating characteristic curve for the diagnosis of sepsis versus SIRS for cf-DNA is 0.60 (0.5 to 0.70). Performance of biomarkers for infection detection was similar in medical patients (*n* = 68) and in surgical patients (*n* = 92).

**Table 2:** Clinical performance of biomarkers in diagnosing sepsis

	**Leukocytes**		**CRP**		**PCT**		**cf-DNA**		**Eosinophils**	
	**Day 1**	**Day 2**	**Day 1**	**Day 2**	**Day 1**	**Day 2**	**Day 1**	**Day 2**	**Day 1**	**Day 2**
Sensitivity (%)	64.96	78.26	90.60	93.91	91.38	81.03	79.31	67.24	64.96	54.87
Specificity (%)	34.88	32.56	40.48	30.95	67.44	67.44	30.23	39.53	28.57	42.50
PPV (%)	73.08	75.63	80.92	78.83	88.33	87.04	75.41	75.00	71.70	72.94
NPV (%)	26.79	35.90	60.71	65.00	74.36	56.86	35.14	30.91	22.64	25.00

cf-DNA, cell-free DNA; CRP, C-reactive protein; NPV, negative predictive value; PCT, procalcitonin; PPV, positive predictive value.

Cutoff values were determined using the Youden index: leukocytes > 11 × 10^9^ cells/l, CRP > 10.38 mg/dl, PCT > 1.39 ng/ml, cf-DNA > 2,850 GE/ml, and eosinophils <25 cells/mm^3^.

#### *Prognostic value*

ICU and hospital mortality rates were 28.1% (45 of 160) and 35.6% (57 of 160), respectively. As shown in Table 1, ICU and hospital mortality rates were not statistically different in these two groups (sepsis vs*.* SIRS without infection).

Comparison between survivors and nonsurvivors is presented in Table 3. APACHE II and SOFA scores were significantly higher in patients who died during hospitalization. The PCT level decreases significantly from day 1 to day 2 in survivors (6.98 ng/ml (31.44) vs*.* 4.06 ng/ml (17.79); *P* < 0.001) but not in nonsurvivors without changes in the other biomarkers. The cutoff values for mortality prediction are CRP > 10.38 mg/dl, PCT > 1.03 ng/ml, cf-DNA > 6,030 GE/ml, and eosinophils < 5 cells/mm^3^. In this case, SOFA score on day 1 presents the greatest AUC (0.69 (95% confidence interval, 0.61 to 0.78)), which is significantly better than the AUC for the rest of the variables (*P* < 0.01).

**Table 3:** Univariate analysis comparing survivors and nonsurvivors in total cohort

	**Total cohort (*n*** **= 160)**	**Survivors (*n*** **= 103)**	**Deaths (*n*** **= 57)**	***P *** **value**
Demographic variables				
Gender (female)	81 (50.6)	58 (56.3)	23 (40.4)	0.053
Age	63 (51 to 74)	62 (51 to 73)	68 (52 to 75)	0.026
Underlying diseases				
Diabetes	35 (21.9)	23 (22.3)	12 (21.1)	0.852
Cirrhosis	6 (3.8)	1 (1)	5 (8.8)	0.022
ESRD	12 (7.5)	7 (6.8)	5 (8.8)	0.756
Immunosuppression	20 (12.5)	11 (10.7)	9 (15.8)	0.349
Cancer	20 (12.5)	7 (6.8)	13 (22.8)	0.003
Chronic cardiac insufficiency	11 (6.9)	6 (5.8)	5 (8.8)	0.523
COPD	22 (13.8)	14 (13.6)	8 (14)	0.938
SIRS				
Two SIRS criteria	41 (25.6)	28 (27.2)	13 (22.8)	0.055
Three SIRS criteria	57 (35.6)	42 (40.8)	15 (26.3)	
four SIRS criteria	62 (38.8)	33 (32)	29 (50.9)	
Severity scores				
APACHE II	17 (12 to 24)	16 (12 to 20)	22 (15 to 27)	<0.001
SOFA	7 (4 to 11)	6 (3 to 9)	9 (6 to 12)	<0.001
Laboratory parameters^a^				
Leukocytes day 1	13,130 (10,185)	13,100 (9,490)	13,160 (12,615)	0.846
Leukocytes day 2	14,700 (8,650)	13,640 (8,290)	17,265 (8,343)	0.014
Eosinophils day 1	55.6 (11.2)	48.34 (9.55)	68.92 (26.51)	0.061
Eosinophils day 2	91.1 (15.6)	81.56 (13.04)	110 (38.99)	0.777
CRP day 1	26.56 (21.91)	27.95 (21.45)	23.44 (22.67)	0.460
CRP day 2	28.85 (19.66)	28.14 (18.10)	31.31 (20.46)	0.377
PCT day 1	7.81 (34.91)	6.98 (31.44)	8.93 (40.28)	0.325
PCT day 2	4.82 (20.56)	4.06 (17.79)	6.69 (26.12)	0.058
cf-DNA day 1	5,265 (5,377)	5,060 (4,760)	6,290 (6,405)	0.730
cf-DNA day 2	4,375 (5,522)	4,480 (6,030)	5,460 (4,580)	0.287
cf-DNA day 1, without ARF^b^	5,050 (5,130)	5,055 (4,562)	5,580 (6,825)	0.812
cf-DNA day 2, without ARF^b^	4,960 (5,410)	4,980 (5,885)	4,570 (5,090)	0.899

Data presented as *n* (%) or median (interquartile range). APACHE, Acute Physiology and Chronic Health Evaluation; ARF, acute renal failure; cf-DNA, cell-free DNA; COPD, chronic obstructive pulmonary disease; CRP, C-reactive protein; ESRD, end-stage renal disease; PCT, procalcitonin; SIRS, systemic inflammatory response syndrome; SOFA, Sequential Organ Failure Assessment. ^a^For comparison, nonparametric tests were used. ^b^ARF defined as renal SOFA ≥3 (*n* = 34).

### Patients with sepsis

#### *Prognostic value*

Table 4 presents the comparison of survivors and nonsurvivors in the 117 patients with sepsis. As occurred in the entire cohort, APACHE II and SOFA scores in the first 24 hours in the ICU were significantly higher in patients who died. On day 1 and day 2, the CRP, PCT or cf-DNA values did not differ between survivors and nonsurvivors. The eosinophil count was higher in nonsurvivors than in survivors, although this difference did not achieve statistical significance (83.41 (35.83) vs*.* 50.13 (11.80); *P* = 0.055). PCT diminished significantly on day 2 in relation to basal levels only in survivors (13.5 (45.26) vs. 8.34 (22.55); *P* < 0.001). The other biomarkers did not significantly change in the two determinations. The APACHE II score exhibits the best AUC as predictor of in-hospital mortality in patients with sepsis (0.690 (0.594 to 0.801)), followed by the SOFA scale (0.630 (0.524 to 0.734)). Comparing patients with severe sepsis and septic shock, only PCT serum levels were significantly higher in patients with septic shock both on days 1 and 2.

**Table 4:** Univariate analysis comparing survivors and nonsurvivors in the septic patient cohort

	**Survivors (*n*** **= 76)**	**Deaths (*n*** **= 41)**	***P *** **value**
Demographic variables			
Gender (female)	39 (51.3)	13 (31.7)	0.042
Age	63 (52 to 73)	66 (52 to 74)	0.301
Underlying diseases			
Diabetes	14 (18.4)	9 (22)	0.647
Cirrhosis	1 (1.3)	4 (9.8)	0.050
ESRD	6 (7.9)	4 (9.8)	0.739
Immunosuppression	10 (13.2)	6 (14.6)	0.825
Cancer	4 (5.3)	11 (26.8)	0.001
Chronic cardiac insufficiency	5 (6.6)	2 (4.9)	0.998
COPD	9 (11.8)	7 (17.1)	0.432
SIRS			
Two SIRS criteria	21 (27.6)	8 (19.5)	0.027
Three SIRS criteria	28 (36.8)	8 (19.5)	
Four SIRS criteria	27 (35.5)	25 (61)	
Severity scores			
APACHE II	16 (12 to 22)	22 (16 to 27)	0.001
SOFA	7 (4 to 11)	10 (6 to 13)	0.007
Laboratory parameters^a^			
Leukocytes day 1	13,035 (9,565)	14,340 (12,730)	0.591
Leukocytes day 2	14,345 (8,208)	17,780 (12,665)	0.010
Eosinophils day 1	50.13 (11.80)	83.41 (35.83)	0.055
Eosinophils day 2	82.23 (14.65)	123.24 (52.62)	0.747
CRP day 1	30.41 (15.77)	23.32 (20.91)	0.101
CRP day 2	29.84 (18.36)	30.72 (20.11)	0.794
PCT day 1	13.50 (45.26)	15.07 (57.42)	
PCT day 2	8.34 (22.55)	12.13 (40.47)	
cf-DNA day 1	5,490 (5,080)	6,660 (7,745)	0.495
cf-DNA day 2	4,830 (6,135)	5,460 (5,400)	0.890
Source of infection			
Surgical	40 (52.6)	29 (70.7)	0.058
Nosocomial	28 (36.8)	16 (39)	0.816
Respiratory	17 (22.4)	8 (19.5)	0.719
Urinary	12 (15.8)	2 (4.9)	0.134
Abdominal	35 (46.1)	18 (43.9)	0.824
Central nervous system	1 (1.3)	0	0.999
Soft tissues	5 (6.6)	4 (9.8)	0.718
Bacteriemic	22 (28.9)	14 (31.4)	0.561
Unknown	3 (3.9)	2 (4.9)	0.999
Others	3 (3.9)	7 (17.1)	0.032
Outcomes			
Length of stay in ICU	8 (4 to 18)	6 (3 to 14)	0.089
Length of stay in hospital	26 (18 to 43)	12 (5 to 22)	<0.001

Data presented as *n* (%) or median (interquartile range). APACHE, Acute Physiology and Chronic Health Evaluation; ARF, acute renal failure; cf-DNA, cell-free DNA; COPD, chronic obstructive pulmonary disease; CRP, C-reactive protein; ESRD, end-stage renal disease; PCT, procalcitonin; SIRS, systemic inflammatory response syndrome; SOFA, Sequential Organ Failure Assessment. ^a^For comparison, nonparametric tests were used.

## Discussion

Our results support the utility of PCT as a biomarker to differentiate sepsis from SIRS without infection. Accuracy of CRP is lower than for PCT, whereas the eosinophil count and cf-DNA serum levels cannot be recommended as indicators of infection in patients with SIRS. In addition, the usefulness of these biomarkers to predict in-hospital mortality is low although a decrease of the PCT levels in the first 2 days foresees a lower mortality.

In clinical routine, the initial differential diagnosis between SIRS and sepsis is sometimes difficult. Clinical signs of infection are nonspecific and the identification of the culprit pathogen is not available in the early hours. Sepsis is associated with a strong acute-phase response resulting in pronounced changes in the concentrations of many plasma components. Apart from their values in discriminating no-sepsis-SIRS from sepsis, several biochemical indicators have been assessed regarding their potential in predicting prognosis.

Regarding the diagnostic utility, PCT was the most useful biomarker for the identification of sepsis. A very recent meta-analysis that assessed the accuracy of PCT as a diagnostic marker of sepsis confirmed its validity and calculated an area under the ROC curve of 0.85 (95% confidence interval, 0.81 to 0.88) [18], very similar to our value. It is also interesting to point out that the cutoff values which separated patients with and without sepsis varied greatly in the different studies. We calculated this value as 1.39 ng/ml, very close to values reported in recent series [19,20].

We have also evaluated PCT as a prognostic biomarker in the cohort of critically ill patients and in the subgroup of septic patients. As in previous studies [21-24], basal PCT determination is not useful to predict mortality and its prediction capacity is lower than the clinical scores (APACHE II or SOFA). We observed a significant decrease of PCT on day 2 with respect to day 1 in survivors that does not occur in nonsurvivors. PCT accuracy for mortality prediction seems to improve with serial measurements [21,25,26]. In septic shock, PCT has been identified as a reliable early prognostic marker in medical patients but not in surgical patients [27].

In agreement with our findings, PCT has been recently identified as a good diagnostic marker but not as a prognostic marker in sepsis [28]. In our study, PCT is significantly higher in septic shock than in severe sepsis. Serum PCT increases with greater severity of sepsis and organ dysfunction [29]. Furthermore, PCT is useful to predict treatment response and as a tool to discontinue antibiotics, a very noteworthy function not evaluated in our research [30].

We observed that the PCT capacity for infection identification is significantly higher than for CRP. PCT is generally considered a more reliable marker than CRP in defining infection as a cause of SIRS [2,3], although other authors concluded that both biomarkers have similar diagnostic accuracy [31,32]. PCT but not CRP (or the other markers) differentiates severe sepsis from septic shock [4]. The different kinetics of these two biomarkers and methodological differences may explain these discrepancies, at least in part.

Different studies have shown that a low eosinophil count is strongly related to the presence of bacterial infections and is associated with a poor prognosis [5,6]. Persistent eosinopenia has also been identified as an independent predictor of mortality in hospitalized patients with bacteremia [33]. In a study that included 68 critically ill patients, diagnosis accuracy of the eosinophil count for sepsis identification was similar to that of CRP or PCT [20]. However, CRP was a better marker than eosinophil count for the diagnosis of bacteremia in critically ill patients [34]. Our results do not support the value of the eosinophil count as a prognostic or diagnostic marker at ICU admission.

Interest has recently developed in the use of cf-DNA as a biomarker in critically ill patients. Little is known about the ability of cf-DNA to differentiate sepsis from other causes of SIRS. Maximum measured concentrations of cf-DNA in the first 4 days in the ICU were significantly higher in infected than in non-infected critically ill patients [12]. In a heterogeneous cohort of 110 febrile patients, concentrations of cf-DNA have a diagnostic capability similar to PCT and higher than that of CRP. Noteworthy is the high AUC of cf-DNA and PCT (0.99 and 0.95 respectively) reported in this study [35].

Moreover, several studies have found that intensive care nonsurvivors present higher cf-DNA concentrations than surviving intensive care patients [11,34-38]. Interestingly, cf-DNA levels do not seem to correlate with CRP or PCT in septic patients [11]. In a cohort of critically ill patients, the maximum measured concentration of cf-DNA in the first 4 days in the ICU but not the levels at admission correlates with mortality and constitutes an independent predictor of in-hospital mortality [12]. In patients with severe sepsis and septic shock, basal cf-DNA has been identified as an independent predictor of ICU mortality but not of hospital mortality [36].

Our results therefore do not support the prognostic usefulness or the discriminative ability of cf-DNA between patients with sepsis or no-sepsis-SIRS – although we excluded patients with a noncure cancer or acute myocardial infarction, conditions that increase cf-DNA levels. Moreover, because cf-DNA is eliminated by urine [39] we excluded patients with renal failure at admission, but the diagnostic or prognostic accuracy of cf-DNA did not improve.

We admit also several limitations. Biomarkers were measured only at admission and 24 hours later. Their performance especially for mortality prediction may be better if the biomarkers were measured daily, although the economic burden would have been clearly greater. The relatively low number of patients included in our study impedes us in exploring the value of these biomarkers in specific infections or in different types or pathogen (i.e. Gram-negative infection). No differences in cf-DNA levels among patients with Gram-positive, Gram-negative, or fungal infections have been reported recently [37]. On the other hand, PCT levels seem to be significantly higher in patients with Gram-negative bacteremia than in bloodstream infections caused by Gram-positive bacteria or fungi [40]. This fact adds uncertainty to the PCT cutoff level that could depend at least in part on the type of pathogen.

Several factors may explain the discrepancies between our results and previous studies regarding the value of cf-DNA. Impaired renal and hepatic function may influence serum cf-DNA levels as cf-DNA crosses the kidney barrier and is partially excreted in urine [39]. The inclusion of patients with derangement of these systems may therefore alter cf-DNA levels. cf-DNA can be measured in serum or in plasma with similar performance [41,42]. We chose serum levels [43] and we consider it very unlikely that this issue could explain differences with studies that determine plasma levels. Very recently, our group has reported that severe traumatic brain injury is associated with elevated serum cf-DNA levels and a decrease during the first 24 hours predicts outcome [9].

## Conclusions

To sum up, the diagnosis of sepsis continues to be a challenge in many clinical situations. PCT at admission to the ICU has a high discriminative power and is superior to CRP or the eosinophil count. cf-DNA levels do not help to identify underlying infections at admission to the ICU of patients with SIRS. Biomarkers evaluated in this study poorly predict outcome although a reduction during the first 24 hours of PCT is associated with a better outcome. Further studies are required to precisely determine the clinical value of cf-DNA in critically ill patients, but our results do not support the incorporation of cf-DNA measurement into clinical practice.

## Key messages

• PCT at admission to the ICU has a high discriminative power of sepsis.

• A reduction of PCT during the first 24 hours is associated with a better outcome.

• cf-DNA levels do not help to identify underlying infections at admission to the ICU of patients with SIRS.

• Biomarkers evaluated in this study poorly predict outcome.

## Abbreviations

APACHE: Acute Physiology and Chronic Health Evaluation; AUC: area under the curve; cf-DNA: cell-free DNA; CRP: C-reactive protein; GE: genome equivalents; PCR: polymerase chain reaction; PCT: procalcitonin; SIRS: systemic inflammatory response syndrome; SOFA: Sequential Organ Failure Assessment.

## Competing interests

The authors declare that they have no competing interests.

## Authors’ contributions

JG-M was responsible for the conception, fund raising, design and coordination of the study, made substantial contributions to data acquisition, analysis and interpretation, and drafted the manuscript. MJH-M, HM and JMG made substantial contributions to the determination of the biomarkers. AG-P made substantial contributions to analysis and interpretation of data. IL, JAM-V and AP-M made substantial contributions to data acquisition and provided useful suggestions. All authors have revised the manuscript and approved the final version of manuscript.
